# NEGATIVE-PRESSURE WOUND THERAPY IN THE TREATMENT OF COMPLEX INJURIES AFTER TOTAL KNEE ARTHROPLASTY

**DOI:** 10.1590/1413-785220172502169053

**Published:** 2017

**Authors:** Camilo Partezani Helito, Daniel Kamura Bueno, Pedro Nogueira Giglio, Marcelo Batista Bonadio, José Ricardo Pécora, Marco Kawamura Demange

**Affiliations:** 1 Universidade de São Paulo, Faculdade de Medicina, Hospital das Clínicas, Instituto de Ortopedia e Traumatologia, Knee Surgery Division, São Paulo, Brazil.

**Keywords:** Negative-pressure wound therapy, Arthroplasty, replacement, knee, Surgical wound dehiscence, Infection

## Abstract

**Objective:**

To present an experience with negative-pressure wound therapy (NPWT) in the treatment of surgical wounds in patients treated for infections after total knee arthroplasty (TKA) with or without dehiscence and prophylaxis in wounds considered at risk of healing problems.

**Methods:**

We prospectively evaluated patients with TKA infection with or without surgical wound dehiscence and patients with risk factors for infection or surgical wound complications treated with Pico^(r)^ device for NPWT in addition to standard treatment of infection or dehiscence in our institution. We considered as an initial favorable outcome the resolution of the infectious process and the closure of the surgical wound dehiscences in the treated cases and the good progression of the wound without complicating events in the prophylactic cases.

**Results:**

We evaluated 10 patients who used Pico^(r)^ in our service. All patients had a favorable outcome according to established criteria. No complications were identified regarding the use of the NPWT device. The mean follow-up of the patients after the use of the device was 10.5 months.

**Conclusion:**

The NPWT can be safely used in wound infections and complications following TKA with promising results. Long-term randomized prospective studies should be conducted to prove its effectiveness. ***Level of Evidence IV, Case Series.***

## RESUMO

**Objetivo:**

Apresentar uma experiência com a terapia com pressão negativa (TPN) no tratamento das feridas cirúrgicas de pacientes tratados por infecções em artroplastias totais do joelho (ATJ) associadas ou não à deiscência e como profilaxia nas feridas consideradas em risco de problemas de cicatrização.

**Métodos:**

Foram avaliados prospectivamente pacientes que apresentavam infecção de ATJ associada ou não à deiscência de ferida operatória e pacientes com fatores de riscos de infecção ou complicações de ferida cirúrgica tratados com dispositivo PICO^(r)^ para TPN além do tratamento padrão da infecção ou deiscência em nossa instituição. Consideramos como desfecho favorável inicial a resolução do processo de infecção e o fechamento das deiscências de ferida operatória nos casos de tratamento e a boa evolução da ferida operatória, sem eventos complicadores, nos casos profiláticos.

**Resultados:**

Foram avaliados 10 pacientes que usaram PICO^(r)^ em nosso serviço. Todos os pacientes apresentaram desfecho favorável de acordo com os critérios estabelecidos. Não foram identificadas quaisquer complicações com relação ao uso do dispositivo de TPN. A média de seguimento dos pacientes após o uso do dispositivo foi de 10 meses e meio.

**Conclusão:**

A TPN pode ser usada em complicações de ferida e infecção depois de ATJ de maneira segura e com resultados promissores. Estudos prospectivos randomizados prolongados devem ser realizados para comprovar sua eficácia. Nível de Evidência IV, Série de Casos.

## INTRODUCTION

Total knee arthroplasty (TKA) is an increasingly common surgery. It is estimated that in 2030 nearly 3.5 million TKA procedures will be performed in the United States.^1^ As the number of arthroplasties increases, the number of complications resulting from this procedure also rises, including surgical wound complications and infection.^2^ Known risk factors for skin complications and infection after TKA include diabetes, obesity, poor nutrition, smoking, and especially prior surgeries.^2^ ^,^ ^3^

Among the measures recommended in the literature to reduce the risk of infection after TKA are the use of prophylactic antibiotics before the incision is made, removing hair with an electric surgical clipper and not a razor, appropriate antisepsis of the hands and forearms, strictly sterile technique, skin preparation with alcohol solution, control of comorbidities such as diabetes and malnutrition in the perioperative period, maintenance of normothermia during the procedure, and appropriate surgical technique which respects the dissection planes.^4^

Once infection is diagnosed, treatment ranges from antibiotics to surgical procedures to clean the wound and remove the surgical implants.^5^ Typically, treatments are long and involve losses in quality of life and function for patients as well as high costs for health services.^6^ ^,^ ^7^

One treatment modality for post-arthroplasty wounds that is becoming more widely known in the orthopedic literature is negative pressure wound therapy (NPWT).^8^ ^,^ ^9^ Although its use is well established in other areas of medicine and orthopedics, particularly in cases of trauma and open fractures, its usage in the field of arthroplasty is not yet well-defined.^8^ ^-^ ^11^

The few studies on these devices in cases of primary arthroplasty do not allow definitive conclusions to be drawn about their use, and despite a theoretical benefit demonstrated by one recent review, no prospective studies clearly demonstrate their benefits.^8^ ^,^ ^9^ Among the possible promising uses for NPWT in arthroplasty are applications in patients at high risk for wound complications, patients with established wound complications, patients with dehiscence or prolonged secretions, and patients with infections. Consequently, more studies are required to investigate each of these clinical situations.

The objective of this present study is to show our experience with NPWT in treating surgical wounds in patients with infections after TKA, associated with or independent of dehiscence, and also as a prophylaxis in wounds considered to be at risk for healing problems.

## METHODS

Two profiles of patients treated in our service were evaluated: patients who presented TKA infection associated with or independent of surgical wound dehiscence, and patients with risk factors for infection or complications of the surgical wound. The study was approved by the institutional ethics board under process number 1247, and all patients in the study signed a consent form.

In the cases of infection, from the time of diagnosis the patients were treated according to the protocol for arthroplasty infection at our institution, which involves antibiotic therapy associated with surgical cleaning and debridement and optional removal of the implant. After the usual treatment, a NPWT device was placed on the wound as an additional measure.

In at-risk patients, the device was immediately installed after the surgical procedure while the patient was still in the surgical suite. The use of this device did not hinder patient participation in the standard rehabilitation they would have received if they did not use the device, since range of motion and gait were stimulated, except when treatment was contraindicated.

**Figure 1:**
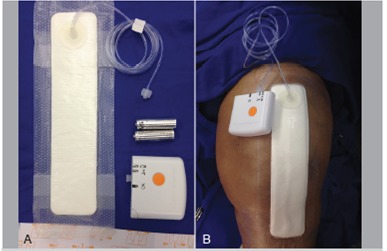
The PICO negative pressure dressing (A) and after application in a patient undergoing surgical cleaning of left knee after arthroplasty (B).

In this study we used a portable single-use PICO device (Smith & Nephew) that applies continuous negative pressure of 80 mmHg.^12^ (Figure 1) After seven days (the working life of the device), we examined the wound and determined whether installation of a new device was necessary. This procedure was repeated every seven days when the device reached the end of its functional life. The total therapy time for each patient was quantified.

We considered resolution of the infection process and closure of dehiscenses in the surgical wound as favorable outcomes in the cases of treatment, and good progress of the surgical wound without complications when this therapy was used prophylactically.

## RESULTS

We assessed 10 patients in our service who used the PICO device. NPWT was indicated in six of these cases for infection, in two cases for infection associated with dehiscence, and two cases in patients at risk. Patient data are summarized in Table 1. Four patients used the device for 14 days (two sessions) and six patients used it for seven days (one session). Mean patient use time was 9.8 days. No patient required NPWT for more than 14 days.

**Table 1:** Summary of assessed patient data.

Patient	Indication for NPWT	Comorbidities	Days NPWT used	Follow-up after use (months)
1	Infection	RA, HBP, DM	14	14
2	Infection	HBP	7	14
3	Prophylactic in at-risk patient	HBP, DM, Obesity	7	13
4	Infection	DM, Obesity	7	12
5	Infection	HBP, DM	7	11
6	Infection	HBP, DM	7	10
7	Infection + dehiscence	HBP, DM	14	10
8	Infection	Gout	14	9
9	Infection + dehiscence	RA	14	9
10	Prophylactic in at-risk patient	HBP, DM, Chagas	7	3 (death)

**Figure 2:**
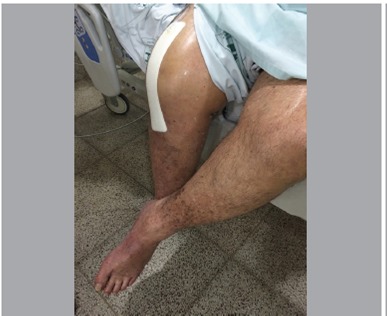
Patient with PICO NPWT dressing on right knee. Note that the dressing does not interfere in range of motion activities during the postoperative period.

As for outcomes, all patients had favorable outcomes according to the criteria: the two patients who received NPWT as a prophylaxis demonstrated healing of the surgical wound without complications, the two cases of dehiscence associated with infection demonstrated closure of the wound and control of the infectious process without the need for surgical intervention, and the six patients who underwent surgery to treat infection showed clinical improvement in infection and good healing. (Figures 2, ^3^, and ^4^).

We did not identify any complications related to the NPWT device. Average patient follow-up time after the use of the device was 10.5 months, ranging from 3 to 14 months. The patient who received three months of follow-up died from causes unrelated to the knee surgery three months after initial treatment, and the wound situation was resolved at that time.

**Figure 3:**
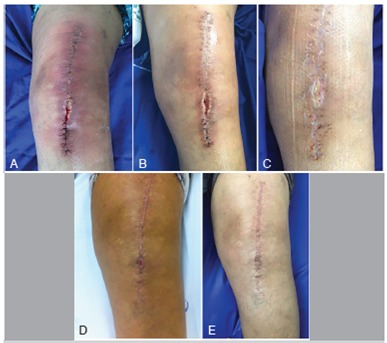
Clinical progress of surgical wound in patient treated with PICO NPWT dressing after dehiscence associated with infection. Photos show initial moment (A) when treatment was indicated and progress at 7 (B), 14 (C), 21 days (D), and 3 months (E). The patient used the dressing for 14 days.

**Figure 4:**
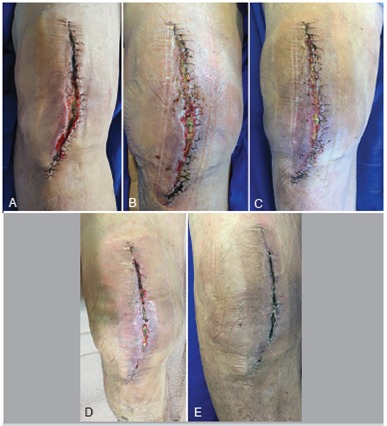
Clinical progress of surgical wound in patient treated with PICO NPWT dressing after dehiscence associated with infection. Photos show initial moment (A) when treatment was indicated and progress at 7 (B), 14 (C), 21 (D), and 30 days (E). The patient used the dressing for 14 days.

## DISCUSSION

The main finding of this study is that NPWT can be used safely to treat post-TKA wound complications and infection without complications and without impeding patient rehabilitation, with promising initial results.

The active mechanisms of NPWT described in the literature which are potentially significant in the use of this therapy in arthroplasty include removal of fluid and reduction of edema, dead space, and soluble inflammatory molecules,^13^ mechanical stabilization, reduction of tension on the wound,^14^ and increased blood flow and angiogenesis.^15^

The potential to control wound complications is significant since this is associated with a great increase in the risk of infection. Patel et al.^16^ ^)^ estimated that each day of persistent drainage represents a 42% increase in the chance of infection, and Saleh et al.^17^ calculated that after the fifth day of secretion, this chance increases 12.7-fold. In a series of 109 cases of hip arthroplasty with persistent post-operatory secretion, Hansen et al.^18^ found that 76% resolved without surgery after negative pressure therapy used for an average of two days (range: 1-10 days). In another series of hip arthroplasties, Pachowsky et al.^19^ observed less seroma in ultrasound in patients who received NPWT. There is no direct relationship between the amount of secretion and post-operatory infection, but patients with prolonged drainage of secretions from the wound (5 or more days post-procedure) have a higher risk of infection.^17^ The decrease in seroma may have been a protective factor for these patients. It is important to note that NPWT should not delay surgical treatment of wound complications or the surgical site in arthroplasties, since as shown by Jaberi et al.,^20^ delayed surgery in the case of secreting wounds leads to an increased risk of failure for treatments involving surgical debridement and irrigation.

In a non-randomized retrospective study, Cooper et al.^21^ compared the use of NPWT with the use of antibiotic dressings in revision arthroplasties of the knee and hip and found fewer wound complications (6.7% versus 26.9%) and fewer instances of infection in the surgical site (3.3% versus 18.5%) with the use of negative pressure therapy.

As for the use of NPWT as an adjunct to treatment of infections in arthroplasty, only small case series have been published,^22^ ^-^ ^24^ showing encouraging results similar to those of this present study.

One randomized study using NPWT in knee arthroplasty had to be halted due to the formation of blisters on the skin surrounding the wound.^25^ Because of this complication, changes were made to avoid blister formation, and this technology was incorporated into the new devices. The PICO NPWT system consists of a multiple-layer silicone dressing designed to avoid the formation of blisters or maceration of the wound.^26^ In our series, we did not observe any complications directly related to NPWT, similar to the other studies which used the same updated device.^26^ ^,^ ^27^

Current contraindications to the use of NPWT include exposed vessels or nerves and unexplored fistulas. Patients with an increased risk of bleeding or who are using anticoagulants should be carefully monitored if they use the device. Circumferential bandages should also be avoided.^9^

Another benefit of the type of therapy used in this study is the possibility of outpatient treatment. Once the dressing is placed, the patient does not require daily dressing changes and the unit can be easily transported. Payne et al.^28^ studied the use of these devices in a wide variety of infections and skin lesions and found a potential cost reduction since patients do not require hospitalization. Dal-Paz et al.^6^ found a significant increase in the costs of treating infections after knee arthroplasty in a tertiary hospital, so that investments in patient safety can bring significant savings to the health system. Matsumoto et al.^27^ concluded that prophylactic NPWT in high-risk patients was reasonable, considering the high costs of treatments resulting from wound complications in arthroplasties.

Although NPWT is widely used in other areas of medicine and is related to improved healing and limb preservation in the treatment of open fractures, it has not yet been proven for use in arthroplasty surgery.^9^ A pilot study conducted by Gillespie et al.^29^ in primary hip arthroplasties suggested that a randomized trial with 900 patients would be required to detect differences in the incidence of complications such as infection, due to the low absolute risk of this complication. Several prospective studies and randomized trials are currently underway to test the efficacy of this therapy.

Despite the small number of cases, the initial results presented in this study are promising. In treating infections, NPWT should be used as an additional tool for patient treatment, and in no way should substitute the gold standard treatments such as antibiotic therapy, surgical treatments, and removal of the implants if necessary.^5^ Our series presented two incidents of dehiscence: one was more superficial and the patient maintained the implant, and the other was deeper since the patient had a cement spacer. Both were successfully treated without the need for a surgical approach. These patients were treated with NPWT for 14 days, since only one dressing was not sufficient. The use of an additional, new dressing should be expected in such high-complexity cases.

Considering eventual recidivism, although these patients were not followed for a long time we focused on healing and skin complications and followed the usual protocols recommended in the literature for treating prosthesis infections, but these cases continue to be monitored for a longer follow-up period in relation to relapse.

Limitations of this study include the small number of patients and short follow-up time, as well as the heterogeneous sample and the absence of a control group. Nevertheless, we believe that the study is important to demonstrate the possible complications and indications of this therapy.

## CONCLUSION

NPWT can be used safely to treat wound complications and infections after knee arthroplasty, with promising results. Long-term prospective randomized studies are still required to prove its effectiveness.
